# Carbonate-Dissolving Bacteria from ‘Miliolite’, a Bioclastic Limestone, from Gopnath, Gujarat, Western India

**DOI:** 10.1264/jsme2.ME11347

**Published:** 2012-03-23

**Authors:** Gangavarapu Subrahmanyam, Ravi Vaghela, Nilesh Pinakinprasad Bhatt, Gattupalli Archana

**Affiliations:** 1 Department of Microbiology & Biotechnology Centre, Faculty of Science, The Maharaja Sayajirao University of Baroda, Vadodara-390002, Gujarat, India; 2 Department of Geology, Faculty of Science, The Maharaja Sayajirao University of Baroda, Vadodara-390002, Gujarat, India

**Keywords:** Carbonate-dissolving bacteria, Limestone ‘miliolite’, 16S rRNA gene sequencing, *Staphylococcus* sp

## Abstract

In the present investigation, the abundance and molecular phylogeny of part of the culturable bacterial population involved in the dissolution of “miliolite”, a bioclastic limestone, from Gopnath, India, was studied. Carbonate-dissolving bacteria were isolated, enumerated and screened for their ability to dissolve miliolite. Amplified ribosomal DNA restriction analysis (ARDRA) indicated 14 operational taxonomic units (OTUs) to be distributed in 5 different clades at a similarity coefficient of 0.85. Then, 16S rRNA sequence analysis helped to decipher that the majority of carbonate-dissolving bacteria were affiliated to phyla *Firmicutes* (Families *Bacillaceae* and *Staphylococcaceae*) and *Actinobacteria* (Family *Promicromonosporaceae*) indicating their role in miliolite weathering.

Biogeochemistry of carbonate minerals is considered to be vital in global carbon cycling, alkalinity generation, and the cycling of major and trace elements among the oceans, continents, and in the atmosphere (6, 32). In the recent past, world-wide attention has been focused on microbially mediated weathering of carbonate rocks, which forms an important link within the exogenic biogeochemical cycle of elements in the karst environment (7, 11, 12, 17, 31, 36). The mechanisms of microbial carbonate weathering include acidolysis, alkaline hydrolysis, complexation, secretion of extracellular polysaccharides and the involvement of enzymes (5, 6, 8, 10). It has been shown that bacteria are able to differentially induce the precipitation or dissolution of carbonates depending on the accessibility of nutrients for their growth (19, 20). Studies show that microbial weathering of rocks is not a simple process of metabolism; instead, it is a process in which microorganisms need to extract one or more limited nutrients from special minerals (2, 11, 33). Therefore, the microbial weathering rate of carbonate rocks depends on trace nutrition components and microbial ecological factors, including their community structure. Cultivation-independent studies of the bacterial community structure of carbonated caves and crusts have been reported earlier (1, 29, 37). These reports indicate that cyanobacteria, proteobacteria, actinobacteria and firmicutes represent the abundant bacteria in carbonated systems; however, several questions remain to be addressed, such as the community structure of carbonate-dissolving bacterial sub-populations, which are crucial for the weathering of carbonates, and the functional role of these bacteria in carbonated environments. Therefore, in the present investigation, for the first time we report a cultural approach to study the carbonate-dissolving bacterial population from a carbonated bioclastic limestone “Miliolite”. The relative abundance, distribution and carbonate-dissolving capability of the isolates are presented on the laboratory scale.

Quaternary carbonate deposits known as miliolites or miliolitic limestone, also referred to as calcarenite (a type of limestone), are deposited in the Saurashtra and Kachchh provinces of Gujarat and appear as patchy outcrop inlands up to 180 km from the miliolite-bearing coast. This limestone derived its name ‘miliolite’ from the foraminifer genus “*Miliolinae*”, which was found abundantly in these deposits (3). Geomorphological, chronological and geochemical studies of these deposits are well established (3). The results show that miliolite deposits predominantly consist of calcite (calcium carbonate [CaCO_3_], mean 86.8%) with aragonite and quartz as minor components. Miliolite clays show the dominance of illite, which is principally generated by the weathering of granite and is brought by the Indus River. Upon weathering, miliolite forms red soil sequences, which are important landscapes in the regions of Saurashtra and Kachchh.

Miliolite and surface red soil sequences studied in the present investigation were collected from the miliolite-bearing natural section in Gopnath (21° 12′ 27.21 N; 72° 06′ 30.08 E), Saurashtra, Gujarat, India. The miliolite-bearing section is pristine in nature and no anthropogenic or animal activities have been noted. Miliolite rocks in the region are weathered and porous. The area is mostly covered with a red soil sequence; however, in many places bare limestone escarpments are present. Triplicate composite miliolitic limestone samples up to a depth of 5 cm were collected using a sterile rock chisel and hammer and were placed in sterile plastic bags in an ice box. Prior to the collection of soil samples, the sites were cleaned by scraping the surface layer up to 1 cm. Triplicate composite red soil samples were collected in sterile plastic bags and immediately placed on ice for transport, as described previously (27). Samples retrieved were denoted M for miliolite rocks and RS for red soil sequences. The soil samples were sieved (<4 mm), cleaned of visible roots and plant residues, and stored at 4°C. Major and minor chemical elements of the miliolite were estimated by an X-ray fluorescence (XRF) spectrometer (30) at the Department of Geology, University of Pune, India. Ca was found to be the most abundant element in miliolite and its proportion was found to be 75.33±6.71%, which is in general agreement with the previous report (3). In view of the fact that miliolite is a bioclastic limestone, a relatively higher amount of organic matter (about 0.8%) was found than in red soil (0.54%).

Since CaCO_3_ is the major mineral phase in miliolite, it was thought that bacteria that have the ability to dissolve carbonate may be keystone species in miliolite weathering. In the present investigation, such species were isolated, characterized and their phylogeny was established. Total viable heterotrophic bacteria (THB) and total carbonate-dissolving bacteria (TCB) were enumerated using R2A medium (26) and Devenze and Bruni (DB) medium (4), respectively (Fig. 1). In brief, miliolite rock was powdered by a sterile mortar and pestle on a clean bench. Miliolite rock powder (10 g) was then homogenized in 90 mL of 0.85% (w/v) NaCl and serially diluted (10 fold) in the same. Aliquots (100 μL) were spread on three independent replicate plates of R2A and DB medium, the plates were incubated aerobically at 30°C for 7 d and the colonies counted at the end of incubation. Similarly, 10 g moist sieved (4 mm) red soil was homogenized in 90 mL of 0.85% (w/v) NaCl, and THB and TCB were enumerated as described above. Bacterial colonies showing a halo zone of clearance around them on DB medium were considered as positive for carbonate dissolution (Fig. 1, inset).

Relatively higher numbers of heterotrophic bacteria were found in red soil (3.1×10^7^, Fig. 1) than in miliolite (6.5×10^4^), whereas a higher number of carbonate-dissolving bacteria (1.7×10^3^, Fig. 1) was noticed in miliolite than in red soil (7.4×10^2^). Moreover, the percentage of the TCB to THB was significantly (*P=*0.001) higher in miliolite (27.17%) than in red soil (0.002%), indicating a higher abundance of carbonate-dissolving bacteria in miliolite than in red soil. This discrepancy is likely due to the geochemistry of the surroundings, since calcium carbonate is a major mineral phase (40–80%) in miliolite; the microorganisms need to dissolve carbonate to extract their nutritional requirements; as a result, high TCB were associated with miliolite. This is in agreement with the understanding that microbial dissolution of carbonate rocks is due to the microorganisms’ limited nutrition that they need from unavailable forms present in minerals (2, 32). In addition, the relatively high amount of organic matter and possibly nitrogen content of miliolite may support the high amount of TCB in miliolite.

Based on the colony size and morphology, 21 carbonate-dissolving bacterial isolates were selected for further analysis. *In vitro* characterization of the miliolite dissolution efficiency of the isolates was determined by measuring the ratio of the zone of clearance (CZ) and colony size (CS) on miliolite agar (reported here for the first time). Miliolite agar is a modified DB medium in which CaCO_3_ is replaced by miliolite powder. The constituents (g L^−1^) of miliolite agar are as follows: glucose, 5 g; yeast extract, 1 g; peptone, 1 g; K_2_HPO_4_, 0.5 g; MgSO_4_, 0.01 g, NaCl, 5 g; NH_4_(SO_4_)_2_, 0.05 g; MgCl_2_, 0.01 g; miliolite rock powder, 5 g and 1.5% agar. For inoculum preparation, bacterial isolates were grown in 3 mL R2A broth for 72 h and 1 mL culture was centrifuged at 12,000×*g* for 5 min and washed three times with 1 mL sterile 0.85% NaCl. The bacterial pellet, resuspended in 1 mL 0.85% NaCl, was used as the inoculum for experiments. A total of 5 μL inoculum (approximately 10^6^ cells mL^−1^) was spotted on miliolite agar plates and incubated aerobically at 30°C for 72 h. Plate assays were replicated three times. The opacity of miliolite agar is due to miliolite rock powder and bacteria that dissolve the miliolite can be distinguished due to the apparent halo of a clear zone around the colony (Fig. 2, inset). At the end of incubation about 14 isolates showed good miliolite dissolution, among which CZ/CS ratios of M16, RS34, M23, M25 and M17 were found to be relatively high (Fig. 2), indicating their *in vitro* efficiency in miliolite weathering.

Amplified ribosomal DNA Restriction Analysis (ARDRA) is a molecular technique widely used to discern the microbial community structure in a range of environments (14, 15, 16, 23, 34). In the present study, the phylogenetc relation among 14 isolates was deciphered by ARDRA performed as follows. DNA was extracted from bacterial isolates (24) and checked for purity and molecular size using conventional agarose gel electrophoresis. Universal eubacterial primers 27F (5′ GAG AGT TTG ATC CTG GCT CAG) and 1107R (5′ GCT CGT TGC GGG ACT TAA CC) were used for the amplification of 16S rRNA gene fragments suitable for ARDRA (21). ARDRA was performed as described previously (18, 25). A binary scoring system (1 for the presence of the band and 0 for its absence) was used to generate an input matrix, which was analysed using UPGMA clustering and subsequent dendrograms. The ARDRA pattern of carbonate-dissolving bacteria (Fig. 3A) revealed that the isolates, depicted as operational taxonomic units (OTUs), were distributed in 5 different clades at a similarity coefficient of 0.85 (Fig. 3B). It was also noted that most of the OTUs were distributed in major evolutionary lineages at a similarity coefficient of 0.54. Around 9 isolates were located in a major lineage whereas minor lineages consisted of 4 isolates.

The 16S rRNA gene fragments amplified by PCR using bacterial genomic DNA as a template were sequenced using commercial sequencing services (Bangalore Genei, Bangalore, India). The best read from the chromatogram was converted to FASTA format. NCBI-BLAST analysis as well as RDP sequence match search was performed. Multiple sequence alignment was carried out in RDP along with representative sequences from the database. A phylogenetic tree was constructed for the isolates using the neighbor-joining method with the MEGA 4.0 program (28). Sequencing results largely corroborated ARDRA results with some variations. It was found that all 14 carbonate-dissolving isolates belonged to phyla *Firmicutes* and *Actinobacteria* (Table S1 and Fig. 4), indicating the prominent role of these taxa in miliolite dissolution. These two phyla were found to be major representatives of the bacterial communities in carbonated environments such as karst and caves, indicating their ability of biomineralization (19, 20). Frozen glycerol stocks of these bacterial strains are maintained at the Department of Microbiology and Biotechnology Centre, The M.S. University of Baroda, India.

Earlier 16S rRNA-based culture-independent studies demonstrated that Gram-negative bacteria belonging to the phylum *Proteobacteria* contributed markedly to the bacterial diversity in carbonated environments (13, 29, 35); however, proteobacterial ability in carbonate dissolution is not yet known. In the present investigation, we did not notice any representative members of *Proteobacteria* which could solubilize carbonate or miliolite. Most of the carbonate-dissolving isolates showed high 16S rRNA gene similarity (98–99%) to *Staphylococcus* sp. (accounting for 57%) and to (96–100%) *Bacillus* sp. (accounting for up to 35.7%). Comparison of CZ/CS ratios (Fig. 2) suggested that *Staphylococcus* species (M16, RS34 and M23) were more proficient at carbonate dissolution than *Bacillus* sp. (M17). The involvement of *Bacillus* sp. in carbonate dissolution is known (9, 10); however, this is the first report indicating the geomicrobial role of *Staphylococcus* sp. in carbonate dissolution. The sole isolate belonging to *Actinobacteria*, showed high 16S rRNA gene identity (100%) to a newly identified (22)*Xylanimonas* sp. in the family *Promicromonosporaceae*.

We conclude that *Firmicutes* and *Actinobacteria* represent two keystone culturable carbonate-dissolving heterotrophic bacterial phyla involved in *in vitro* carbonate dissolution and attendant miliolite weathering. The mechanisms of these isolates in miliolite weathering in the natural environment are presently under investigation. Although the present investigation deals specifically with the culturable bacterial population, it does not preclude the possibility of as yet uncultivable carbonate-dissolving bacteria for which we do not have any information. Application of molecular tools and isotope techniques may help to extend the knowledge about total carbonate-dissolving microbial communities and their specific function *in situ*. The present study improves our conceptual understanding of microbial communities as important players in carbonate weathering, which has a wide range of implications; from the elucidation of biogeochemical cycles to the potential impact of atmospheric CO_2_ sinks (12).

Nucleotide sequences retrieved from this study have been submitted to Genbank under accession numbers JN092561 to JN092574.

## Supplementary Material



## Figures and Tables

**Fig. 1:**
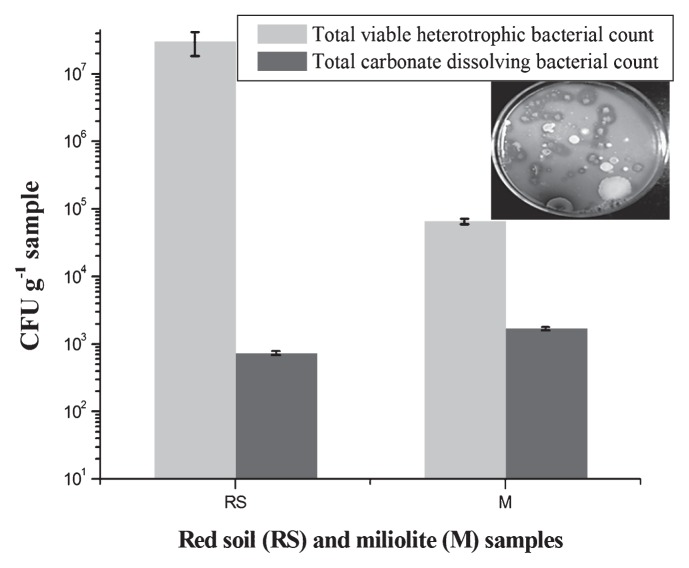
Enumeration of total viable heterotrophic bacteria (THB) and total carbonate-dissolving bacteria (TCB) from miliolite (M) and weathered red soil (R). The results are shown for three independent measurements and error bars depict S.D. A representative plate with carbonate-dissolving bacterial colonies on DB medium is shown in the inset. Clear halo zone around the bacterial colony indicates carbonate solubilization.

**Fig. 2:**
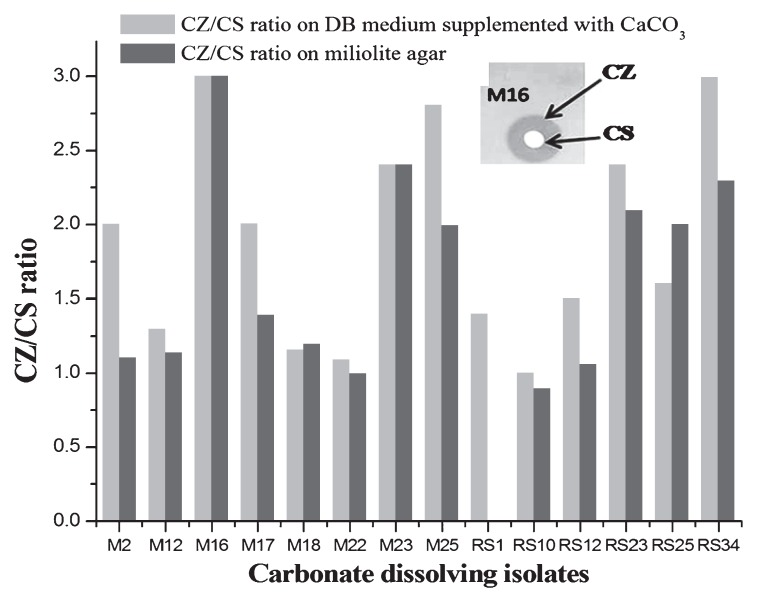
Zone of clearance (CZ)/colony size (CS) ratios of carbonate-dissolving bacteria on DB medium and miliolite agar. Inset shows CZ and CS of one of the isolates (M16) on miliolite agar.

**Fig. 3:**
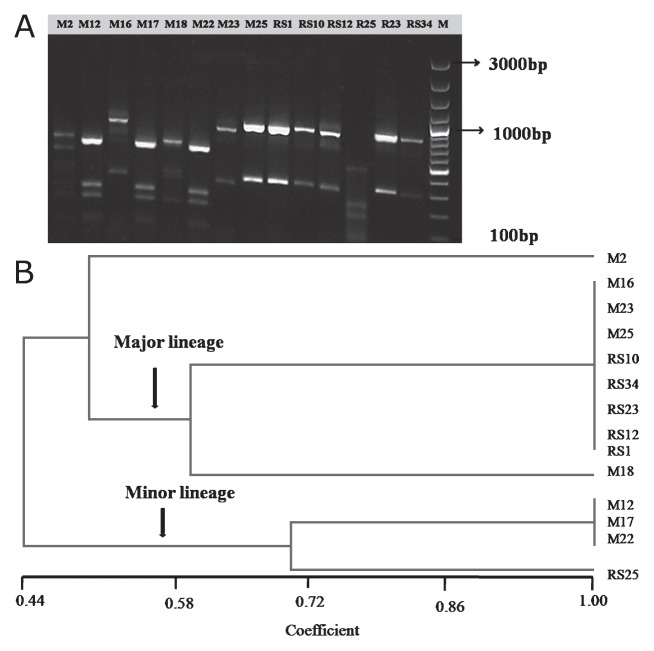
ARDRA pattern of the carbonate-dissolving bacteria (A) and their phylogenetic relationship (B). M indicates miliolite isolates, whereas RS indicates red soil isolates.

**Fig. 4:**
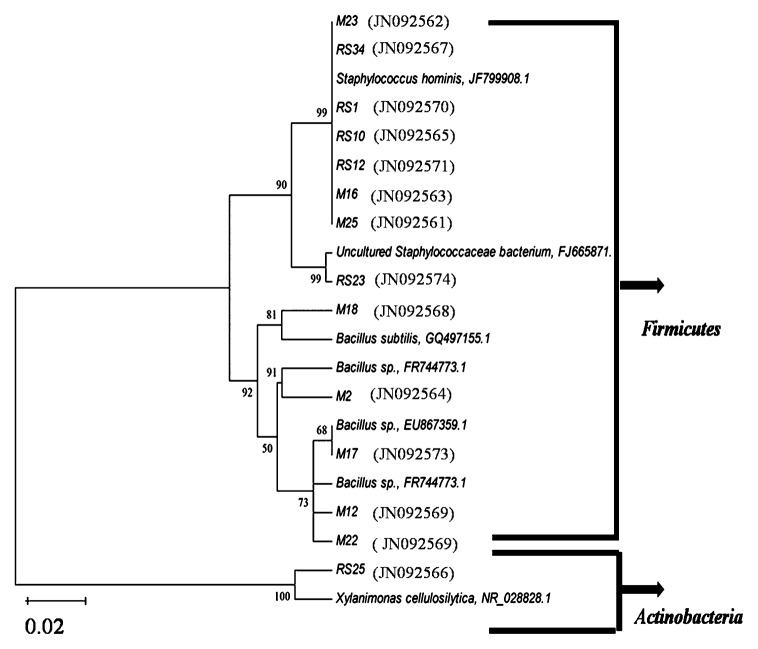
16S rRNA gene sequence-based phylogenetic tree of carbonate-dissolving bacteria. The evolutionary history was inferred using the UPGMA method. Phylogenetic analysis was conducted using MEGA4 (28). M indicates miliolite isolates, whereas RS indicates red soil isolates. Genbank accession numbers of the sequences reported in this study are shown in parentheses.
