# Supplementary material for: Carbonate-Dissolving Bacteria from ‘Miliolite’, a Bioclastic Limestone, from Gopnath, Gujarat, Western India
Source: Microbes Environ. 2012 Mar 23;27(3):334–7. doi: 10.1264/jsme2.ME11347 (PMC4036042; doi:10.1264/jsme2.ME11347)
Supplement: Supplementary file 1 [file 27_334_s1.pdf]

Table S1. 16S rRNA gene sequence similarity of miliolite-dissolving bacteria. Isolates designated with suffix M were obtained from miliolite sample and RS, from red soil.

Isolate	Genbank accession numbe	Closest match in Genbank	% Similarity	Genbank accession number of closest matc	Eubacterial Phylu	Eubacterial Family
M25	JN092561	*Staphylococcus hominis*	99	JF799908.1	*Firmicutes*	*Staphylococcaceae*
M23	JN092562	*Staphylococcus hominis*	98	JF799908.1	*Firmicutes*	*Staphylococcaceae*
M16	JN092563	*Staphylococcus hominis*	99	JF799908.1	*Firmicutes*	*Staphylococcaceae*
M2	JN092564	*Bacillus* sp.	96	DQ275174.1	*Firmicutes*	*Bacillaceae*
RS10	JN092565	*Staphylococcus hominis*	99	JF799908.1	*Firmicutes*	*Staphylococcaceae*
RS25	JN092566	*Xylanimonas cellulosilytica*	100	NR_028828.1	*Actinobacteria*	*Promicromonosporaceae*
RS34	JN092567	*Staphylococcus hominis*	99	JF799908.1	*Firmicutes*	*Staphylococcaceae*
M18	JN092568	*Bacillus subtilis*	100	GQ497155.1	*Firmicutes*	*Bacillaceae*
M12	JN092569	*Bacillus* sp.	100	FR744773.1	*Firmicutes*	*Bacillaceae*
RS1	JN092570	*Staphylococcus hominis*	99	JF799908.1	*Firmicutes*	*Staphylococcaceae*
RS12	JN092571	*Staphylococcus hominis*	99	JF799908.1	*Firmicutes*	*Staphylococcaceae*
M22	JN092572	*Bacillus* sp.	100	FR744773.1	*Firmicutes*	*Bacillaceae*
M17	JN092573	*Bacillus* sp.	100	EU867359.1	*Firmicutes*	*Bacillaceae*
RS23	JN092574	Uncultured Staphylococcaceae bacterium	100	FJ665871.1	*Firmicutes*	*Staphylococcaceae*
